# Patisiran for the treatment of patients with p.Ile88Leu hereditary transthyretin amyloidosis: an Italian real-life experience

**DOI:** 10.3389/fneur.2024.1415851

**Published:** 2024-06-07

**Authors:** Giacomo Urbinati, Ilaria Cani, Marco Currò Dossi, Simone Longhi, Samuela Carigi, Christian Gagliardi, Elena Biagini, Nazzareno Galiè, Pietro Cortelli, Pietro Guaraldi

**Affiliations:** ^1^Dipartimento di Scienze Biomediche e Neuromotorie (DIBINEM), Alma Mater Studiorum Università di Bologna, Bologna, Italy; ^2^IRCCS Istituto delle Scienze Neurologiche di Bologna, Bologna, Italy; ^3^Neurology Unit, Ospedale degli Infermi, Rimini, Italy; ^4^Cardiology Unit, Cardiac Thoracic and Vascular Department, IRCCS Azienda Ospedaliero-Universitaria di Bologna, Bologna, Italy; ^5^Cardiology Unit, Ospedale degli Infermi, Rimini, Italy

**Keywords:** hereditary transthyretin amyloidosis, patisiran, p.Ile88Leu, TTR, familial amyloid polyneuropathy, transthyretin amyloid cardiomyopathy

## Abstract

**Objectives:**

Evidence on the activity of patisiran therapy in specific subgroups of patients with hereditary transthyretin amyloidosis variant (ATTRv) is still scarce. This prospective real-world study was designed to provide the first in-depth clinical data on the effectiveness of patisiran in patients with ATTRv reporting the p.Ile88Leu variant, the most widespread variant in the Emilia-Romagna regional area, which has been less represented in previous clinical trials.

**Patients and methods:**

This prospective study evaluated all the patients with genetically proven ATTRv (p.Ile88Leu) and polyneuropathy treated with patisiran in the Emilia-Romagna referral centers for ATTRv (Institute of Neurological Sciences in Bologna and Division of Neurology in Rimini) from March 2021 to April 2023. All subjects underwent clinical and neurological evaluations at baseline and after 9–12 months of treatment.

**Results:**

A total of 22 patients were included in the study; the median age was 73 years (IQR: 9), the age at diagnosis was 72 years (IQR: 10), and the disease duration was 1.6 years (IQR: 2.3). We observed stability of all considered neurological and cardiological parameters at 9–12 months after the beginning of patisiran treatment.

**Conclusion:**

Our findings support the clinical data regarding the effectiveness of patisiran in stabilizing the disease course and extend this activity to the subset of patients with the p.Ile88Leu variant.

## Introduction

Hereditary transthyretin amyloidosis (ATTRv) is a rare, autosomal dominant, multisystem disease caused by mutations in the transthyretin (TTR) gene (1). The main features of the disease are progressive sensory-motor and autonomic neuropathy and cardiomyopathy (2, 3).

ATTRv is a rare disease; Portugal and Sweden are considered endemic areas, and Japan, Brazil, Maiorca, and Cyprus have been reported as other foci (4).

Recent progress in the diagnosis points out that more patients with ATTRv are affected worldwide than expected. Specifically, more than 140 missense mutations in the *TTR* gene have been recognized to date, and significant regional variations in genotype and phenotype distribution have been observed (5, 6). Regarding the Italian scenario, the national prevalence of ATTRv is 4.33/million, with higher rates in southern Italy and in Lazio region (in particular, in this latter area, the prevalence is around 17.2/million) (7); the most common variant in patients and asymptomatic carriers is p.Ile88Leu, which is particularly frequent in Emilia Romagna and Tuscany and Romagna Apennines (8).

Little is known about most ATTR variants (9). Concerning p.Ile88Leu, although it is mainly associated with a cardiac phenotype, frequent neurological symptoms have been reported in affected patients, including carpal tunnel syndrome (reported in 43% of patients), which may precede the diagnosis of amyloidotic cardiomyopathy within 5–7 years, autonomic dysfunction and sensory-motor distal polyneuropathy (10, 11). The natural history of this variant has been explored in a recent study by our group (12) and is characterized by high mortality in the short term (41% at 3 years and 63% at 5 years) (11).

Over the past 15 years, increasing knowledge of the molecular mechanisms of the disease has enabled the progressive development of new specific disease-modifying therapies to slow its progression (13). Among them, patisiran is a small, double-stranded interfering RNA that selectively targets TTR mRNA, reducing both ATTRv and wild-type ATTR production (14, 15). The phase III placebo-controlled APOLLO study evaluated the efficacy and safety of patisiran versus placebo at 18 months in 225 patients (15). Within the APOLLO study, patisiran showed the ability to preserve functional capacity, health status, and quality of life (QoL) in patients with ATTRv and polyneuropathy regardless of the stage of the disease, the associated TTR variant, and the age of onset. In contrast, placebo was associated with steady worsening (15). In detail, improvements compared with placebo were reported in the modified Neuropathy Impairment Score + 7 (mNIS+7, primary endpoint: 56% vs. 4%), Norfolk Quality of Life-Diabetic Neuropathy (Norfolk QOL-DN, 51.4% vs. 10.4%), gait speed (53% vs. 13%) and Composite Autonomic Symptom Scale-31 (COMPASS-31) measure of autonomic symptoms (15). These results were confirmed by the global OLE study, demonstrating that patisiran could maintain long-term efficacy (16). Furthermore, an analysis of several cardiac parameters in a pre-specified cardiac subpopulation of APOLLO study showed a beneficial effect on cardiomyopathy, suggesting that patisiran could halt or reverse the progression of cardiac symptoms of patients with ATTRv (17).

Overall, 39 TTR variants were reported among the patients enrolled in the APOLLO study; however, no patient had the p.Ile88Leu variant (15). Similarly, within the APOLLO-B study, only one patient in the treatment group reported the p.Ile88Leu variant (17). Consequently, evidence on the activity of patisiran therapy in this specific subgroup of patients is still scarce. The study aimed to provide additional knowledge on the effectiveness of patisiran treatment in real-world practice, considering a p.Ile88Leu patient population. In particular, this prospective study analyzed the activity of patisiran in patients from Emilia-Romagna affected from p.Ile88Leu ATTRv and polyneuropathy, monitoring both neurological and cardiological parameters.

## Patients and methods

In this study, we prospectively evaluated all the patients with genetically proven ATTRv (p.Ile88Leu) and polyneuropathy treated with patisiran in the Emilia-Romagna referral centers for ATTRv (Institute of Neurological Sciences in Bologna and Division of Neurology in Rimini) from March 2021 to April 2023. Patisiran was administered as an intravenous infusion at 300 μg/kg dose every 3 weeks.

All the subjects underwent clinical and neurological evaluations at baseline (T0) and after 9–12 months of treatment (T1, range varies depending on the latest available clinical assessment), including a Familial Amyloid Polyneuropathy (FAP) scale (with higher scores indicating more impaired walking ability), Neuropathy Impairment Score (NIS) (range, 0–192, with higher scores indicating more impairment), COMPASS-31 scale score (for evaluation of autonomic dysfunction symptoms), a QoL assessment with the Norfolk QOL-DN and Karnofsky Performance Status Scale questionnaires (range − 4–136 and 0–100, respectively, with higher scores indicating worse QoL). Patients also underwent extensive cardiological evaluation at both T0 and T1, including cardiological examination, transthoracic echocardiogram, and blood tests. Statistical analysis was conducted using SPSS software (29.0 version). We focused on indicators readily available in clinical practice that do not need dedicated instruments (i.e., QST). Data were expressed as median (interquartile range [IQR]). T0 and T1 data were compared with the Wilcoxon signed-rank test.

The study was conducted in accordance with the ethical principles of the revised version of the Declaration of Helsinki (52nd WMA General Assembly, Edinburgh, Scotland, October 2000) and with the protocol approved by the Ethics Committee of the Local Health Authority of Bologna (reference number CE21006, P.I. Dr. P. Guaraldi). All patients gave consent to the publication of their clinical data for scientific and educational purposes.

## Results

A total of 22 patients were included in the study; the median age was 73 years (IQR: 9), the age at diagnosis was 72 years (IQR: 10), and the duration of disease was 1.6 years (IQR: 2.3). Further clinical features were reported in Table 1. As with previous therapies, three patients (14%) were treated with tafamidis, two patients (9%) had combined heart and liver transplants and one patient had liver transplant alone.

**Table 1:** Patient baseline characteristics.

Features	n (%)/median (IQR)
Males	18 (82%)
Age (years)	73 (9)
Age at diagnosis (years)	72 (10)
Weight (kg)	69.5 (17.0)
BMI	24.6 (5.4)
Duration of disease (years)	1.6 (2.3)
Previous therapies	
Tafamidis	3 (13.6%)
Liver transplant	3 (13.6%)
Cardiac transplant	2 (9.1%)
Perugini score*	
2	12 (54,5%)
3	7 (31.8%)
NIS score	22 (23)
COMPASS-31 scale	18 (30)
Norfolk quality of life	36 (40)
Karnofsky performance status scale	80 (10)
NYHA class	2 (2)
Left ventricle wall thickness (mm)	16.5 (4.9)
Left ventricle ejection fraction (%)	53 (18)
Left ventricle telediastolic volume (mL)	86 (40)
NT-ProBNP (pg/mL)	1,300 (2795)

*Available for 19 patients. NIS, Neuropathy Impairment Score; NYHA, New York Heart Association; NT-ProBNP, N-terminal pro–B-type natriuretic peptide.

Cardiac amyloidosis evaluation with ^99m^Tc-DPD scintigraphy was available for 19 patients; most had a Perugini score of 2 (12 patients, 54.5%); regarding the three patients for whom the scintigraphy was not reported, two of them received combined heart and liver transplantation and were confirmed by biopsy. In the other case, the diagnosis was made considering the high suggestiveness of the ECG and echocardiographic findings, combined with the genetic evidence of the mutation and the absence of other possible compatible causes (particularly the absence of blood dyscrasias).

Neurological evaluation at baseline documented 17 patients (77%) with polyneuropathy FAP stage 1 and five patients (23%) with polyneuropathy FAP stage 2. Overall, the median NIS score was 22 (IQR: 23), and the COMPASS-31 scale score was 18 (IQR: 30). Norfolk QOL-DN median score was 36 (IQR: 40), and the Karnofsky Performance Status Scale median score was 80 (IQR: 10) (Table 1).

Baseline cardiological features were characterized by a median New York Heart Association (NYHA) stage 2 (IQR: 2), mean left ventricle wall thickness of 16.5 mm (IQR: 4.9), left ventricle ejection fraction of 53% (IQR: 18%), left ventricular telediastolic volume of 86 mL (IQR: 40), and N-terminal pro–B-type natriuretic peptide of 1,300 pg./mL (IQR: 2,795).

### Follow-up parameters

**Figure 1:**
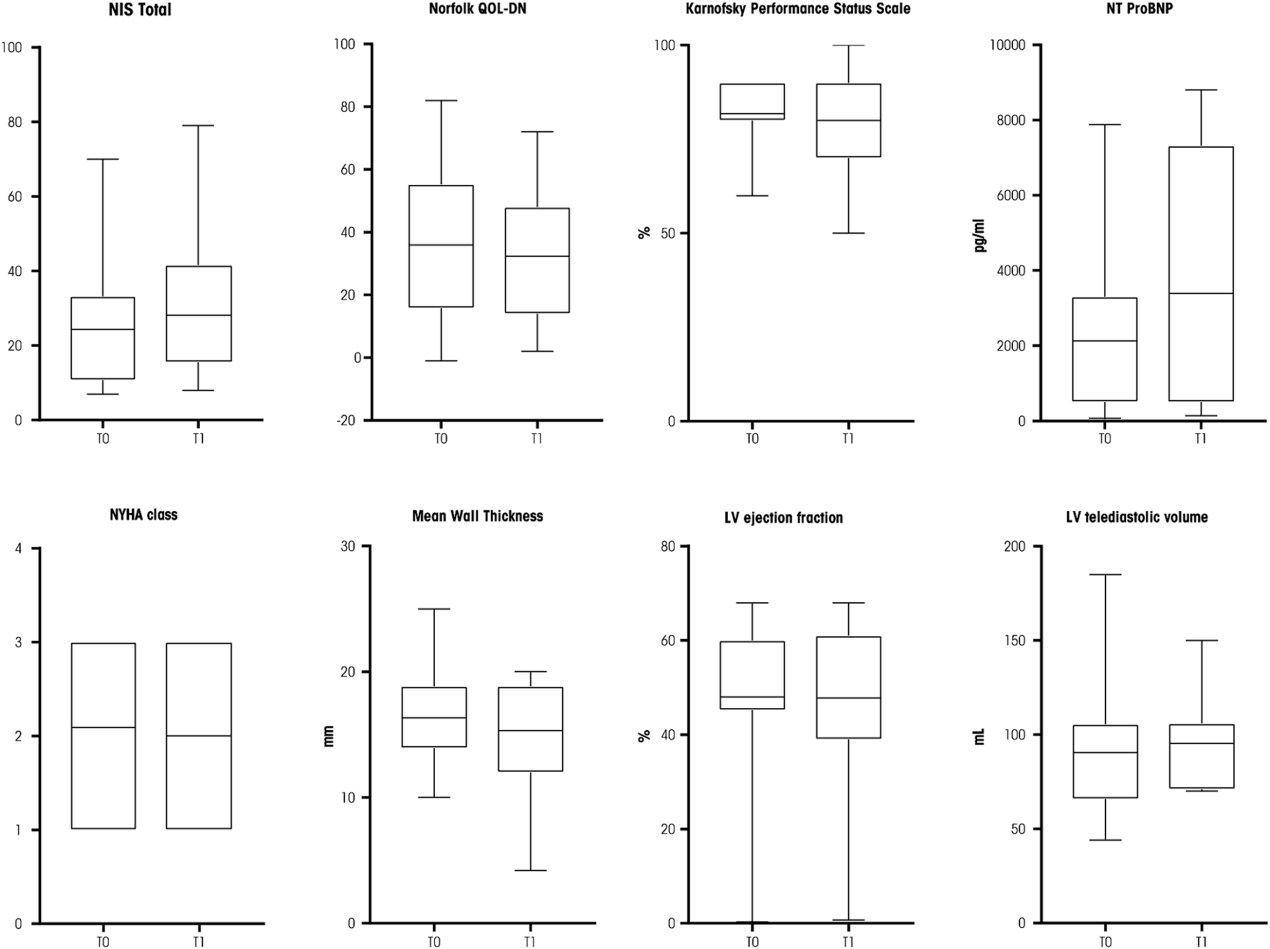
Box plots of neurological and cardiological parameters assessed at T0 and T1. NIS, Neuropathy Impairment Score; NT-ProBNP, N-terminal pro-B-type natriuretic peptide; NYHA, New York Heart Association; LV, left ventricle. Source: original.

Neurological and cardiological parameters stability was observed after 9–12 months at T1 compared with T0 values (Figure 1). In detail, a median NIS total scale of 20 (IQR: 26; *p* = 0.698), Norfolk QOL-DN of 30 (IQR: 34; *p* = 0.618), and a Karnofsky Performance Status Scale of 90 (IQR: 20; *p* = 0.527) were reported. The FAP stage at T1 was unchanged (5/22 patients had polyneuropathy stage 2, the others stage 1).

The cardiological parameters also proved to be stable at T1 (Figure 1): median NYHA class was 2 (IQR: 2; *p* = 0.999), mean left ventricle wall thickness was 15.3 mm (IQR: 6.9; *p* = 0.672), left ventricle ejection fraction was 52% (IQR: 22; *p* = 0.799), and left ventricular telediastolic volume was 96 mL (IQR: 80; *p* = 0.138). A trend for an increased serum level of N-terminal pro–B-type natriuretic peptide was reported (2,130 pg./mL, IQR, 6,825, *p* = 0.050, Figure 1).

During the follow-up, two patients were hospitalized: one patient was admitted for a hip fracture following an accidental fall, while a second patient was admitted twice for heart failure and eventually died from voluntary ingestion of drugs.

In our cohort, only one patient experienced adverse reactions to the medication, consisting of an urticarial reaction treated with antihistamines with resolution of the reaction. During subsequent infusions, the patient underwent premedication with antihistamines starting 2 days before, in addition to the standard premedication, with no further adverse reactions.

## Discussion

Patisiran is an effective therapy for neurological and cardiac parameters in patients with ATTRv and polyneuropathy stage 1 or 2, as previously demonstrated in the pivot clinical trial APOLLO (15).

Our prospective real-world study provided for the first time in-depth clinical data on the effectiveness of patisiran in patients with ATTRv reporting the p.Ile88Leu variant, the most widespread mutation in our Italian regional area, which has been less represented in previous clinical trials (15, 17). We observed the stability of all the considered neurological and cardiological parameters at 9–12 months after the initiation of the treatment with patisiran. These findings support the clinical data regarding the effectiveness of patisiran in stabilizing the disease course and extend this activity to the subset of patients with the p.Ile88Leu variant.

Among the available disease-modifying therapies, vutrisiran was recently approved. Similar to patisiran, it is an RNA interference therapeutic that reduces the synthesis of variant and wild-type TTR (18). Vutrisiran siRNA–N-Acetylgalactosamine (GalNAc) is characterized by enhanced stabilization chemistry leading to increased potency and high metabolic stability, allowing subcutaneous injection once every 3 months. Consistent with the pharmacodynamic effects observed in the phase I vutrisiran study (18), vutrisiran and patisiran demonstrated comparable effectiveness in treating ATTRv with polyneuropathy at 9 and 18 months within the HELIOS-A study. The patterns of stabilization or improvement across endpoints observed in the APOLLO study were also observed in the HELIOS-A trial (19, 20). Although vutrisiran and patisiran use different siRNA delivery platforms (vutrisiran is conjugated to a triantennary GalNAc ligand that binds the asialoglycoprotein receptor expressed on the surface of hepatocytes), serum TTR reduction was comparable, resulting in consistent clinical benefits. Therefore, we expect similar results in the real-world experience with vutrisiran as with patisiran in the p.Ile88Leu patient cohort.

Our study’s limitations are the small sample size and the short follow-up period due to the rarity of this condition. We did not analyze many biomarkers typically assessed in clinical studies, such as Quantitative Sensory Testing (QST), autonomic parameters, intraepidermal nerve fiber density (IENFD), composite scores like mNIS+7, or scales like the time-up-and-go (TUG) or 6-min walking test (6MWT). These tests are not part of our clinical routine and were not included as they extend beyond the scope of our study’s real-life context. Therefore, further studies with a larger sample and longer follow-ups are warranted to confirm our results.

## Data availability statement

The original contributions presented in the study are included in the article/supplementary material, further inquiries can be directed to the corresponding author.

## Ethics statement

The studies involving humans were approved by the Ethics Committee of the Local Health Authority of Bologna (reference number CE21006, P.I. Dr. P. Guaraldi). The studies were conducted in accordance with the local legislation and institutional requirements. The participants provided their written informed consent to participate in this study.

## Author contributions

GU: Data curation, Formal analysis, Writing – original draft, Writing – review & editing. IC: Data curation, Formal analysis, Writing – original draft, Writing – review & editing. MCD: Conceptualization, Data curation, Formal analysis, Writing – review & editing. SL: Data curation, Formal analysis, Writing – review & editing. SC: Data curation, Formal analysis, Writing – review & editing. CG: Data curation, Formal analysis, Writing – review & editing. EB: Data curation, Formal analysis, Writing – review & editing. NG: Data curation, Formal analysis, Writing – review & editing. PC: Conceptualization, Data curation, Formal analysis, Writing – review & editing. PG: Conceptualization, Data curation, Formal analysis, Writing – original draft, Writing – review & editing.
